# Older adults must hurry at pedestrian lights! A cross-sectional analysis of preferred and fast walking speed under single- and dual-task conditions

**DOI:** 10.1371/journal.pone.0182180

**Published:** 2017-07-31

**Authors:** Patrick Eggenberger, Sara Tomovic, Thomas Münzer, Eling D. de Bruin

**Affiliations:** 1 Institute of Human Movement Sciences and Sport, Department of Health Sciences and Technology, ETH Zurich, Zurich, Switzerland; 2 Institute of Physiotherapy, School of Health Professions, Zurich University of Applied Sciences, Winterthur, Switzerland; 3 Geriatrische Klinik St.Gallen, St.Gallen, Switzerland; 4 Department of Epidemiology, CAPHRI School for Public Health and Primary Care, Maastricht University, Maastricht, Netherlands; 5 Centre for Evidence Based Physiotherapy, Maastricht University, Maastricht, Netherlands; University of Akron, UNITED STATES

## Abstract

Slow walking speed is strongly associated with adverse health outcomes, including cognitive impairment, in the older population. Moreover, adequate walking speed is crucial to maintain older pedestrians’ mobility and safety in urban areas. This study aimed to identify the proportion of Swiss older adults that didn’t reach 1.2 m/s, which reflects the requirements to cross streets within the green–yellow phase of pedestrian lights, when walking fast under cognitive challenge. A convenience sample, including 120 older women (65%) and men, was recruited from the community (88%) and from senior residences and divided into groups of 70–79 years (*n* = 59, 74.8 ± 0.4 y; mean ± SD) and ≥80 years (*n* = 61, 85.5 ± 0.5 y). Steady state walking speed was assessed under single- and dual-task conditions at preferred and fast walking speed. Additionally, functional lower extremity strength (5-chair-rises test), subjective health rating, and retrospective estimates of fall frequency were recorded. Results showed that 35.6% of the younger and 73.8% of the older participants were not able to walk faster than 1.2 m/s under the fast dual-task walking condition. Fast dual-task walking speed was higher compared to the preferred speed single- and dual-task conditions (all *p* < .05, *r* = .31 to .48). Average preferred single-task walking speed was 1.19 ± 0.24 m/s (70–79 y) and 0.94 ± 0.27 m/s (≥80 y), respectively, and correlated with performance in the 5-chair-rises test (*r*_*s*_ = −.49, *p* < .001), subjective health (τ = .27, *p* < .001), and fall frequency (τ = −.23, *p* = .002). We conclude that the fitness status of many older people is inadequate to safely cross streets at pedestrian lights and maintain mobility in the community’s daily life in urban areas. Consequently, training measures to improve the older population’s cognitive and physical fitness should be promoted to enhance walking speed and safety of older pedestrians.

## Introduction

In the older population, reduced usual or preferred walking speed is strongly associated with increased risk for disability, cognitive impairment, falls, and all-cause mortality [1, 2]. A recent meta-analysis demonstrated that the risk of all-cause mortality is elevated by 89% in the older adults exhibiting the lowest preferred walking speeds [1]. Walking speed was therefore proposed as a simple geriatric assessment to identify older adults with increased mortality risk and was suggested to be “the sixth vital sign” [1–6]. The increase in all-cause mortality risk in relation to slow walking speed is mostly associated with increased cardiovascular mortality risk [7, 8]. Moreover, reduced walking speed is predictive of community functioning [9] and is an issue for older adults attempting to maintain their mobility and safety as pedestrians in urban areas [10]. In contrast to what intuitively might be assumed, aging itself is not a strong explanatory factor for the observable slowing of preferred gait speed in the older population. Healthy older adults walk at a speed that exceeds standards for crossing urban streets; furthermore, they are able to adopt a significantly faster gait in response to a crosswalk signal albeit this comes at the cost of increased gait variability [11]. It seems that especially potentially modifiable factors such as impairment of Instrumental Activities of Daily Living, physical inactivity and cardiovascular disease, are related to the observed slowing of walking speed in many older adults [12].

*Preferred walking speed* of a substantial proportion of older adults is slower than 1.2 m/s [13–17]. This speed is required, however, at pedestrian lights in Switzerland and many other countries, including the United States, Canada, the United Kingdom, Ireland, and South Africa, to cross streets safely within the green–yellow phase [11, 13, 14, 18, 19]. For instance, a recent comprehensive investigation from Ireland reported that 31% of older adults in the age group of 65–74 years and 61% of those older than 75 years were walking slower than 1.2 m/s at their preferred speed [13]. Another study, from the United Kingdom, found that up to 84% of men and 93% of women older than 65 years of age did not reach 1.2 m/s with normally paced walking [14]. Recent research indicates that older pedestrians’ mobility and safety is not only negatively affected by physical frailty, which is characterized by slow walking speed, low physical activity, unintentional weight loss, exhaustion, and muscle weakness [20], but also by attention deficits (and visual impairment) [10]. This finding is supported by other investigations linking executive control and attention deficits with increased fall risk [21, 22]. Thus, it appears questionable if the measurement of single-task preferred walking speed adequately reflects the physical and cognitive requirements that are co-existing when crossing a street [23, 24].

Several studies support the notion that *cognitive–motor dual-tasking* and divided attention play an important role in street crossing behavior of older adults. For instance, older adults were more susceptible to dual-task impairments compared to younger adults in a simulated virtual reality street crossing task. In contrast, younger adults did mostly not show any dual-task costs from listening to music or talking on a cell phone while street crossing [24]. Dual-task costs are referred to as percentage of loss, relative to the single-task street crossing or walking condition [24, 25]. Previous research has shown that high dual-task costs for different gait parameters, including stride time, stride velocity and stride length at fast walking speed, are associated with poorer divided attention performance [26]. In addition, a divided attention experiment on a simulated street demonstrated that older adults with poorer perceptual, physical, and cognitive functions were prone to make risky street crossing decisions [27]. Finally, another simulation study identified several predictors of dangerous street-crossing choices which included low walking speed, as well as visual processing speed, visual attention, and attention shifting ability [28]. Therefore, measuring dual-task walking speed, including a cognitive task, may represent a better approximation of real-life conditions for crossing streets at pedestrian lights.

To the best of our knowledge, to date only one study has assessed *preferred dual-task walking speed* as a measure to estimate the proportion of older adults that is at risk when crossing streets at pedestrian lights [13]. The authors demonstrated that the percentage of older adults (≥75 years of age) walking slower than 1.2 m/s at preferred speed rose from 61% under single-task to 91% under dual-task walking conditions, respectively [13]. Furthermore, a recent systematic review identified the lack of research examining the effect of cognitive dual-tasks on gait speed in community-dwelling older adults [29]. Particularly, studies are inexistent that include a *fast speed dual-task walking* condition which might reflect time pressure and cognitive challenge when crossing streets at pedestrian lights even more appropriately [26].

Therefore, this study aimed to identify in a convenience sample of Swiss older adults the proportion that doesn’t reach 1.2 m/s walking speed, which is required to safely cross streets within the green–yellow phase of pedestrian lights, while walking fast under a cognitively challenging dual-task condition. We hypothesized, that under this *fast speed dual-task walking* condition the proportion of older adults walking slower than 1.2 m/s would be smaller compared to the *preferred speed single- and dual-task walking* conditions.

## Materials and methods

### Study design and participants

This study represents a cross-sectional evaluation of preferred and fast walking speed under single- and dual-task conditions among 120 older adults living in a Swiss city with about 80’000 residents [30]. We used baseline gait data of 90 older adults enrolled for a six-month training intervention as described previously [25, 31]. In addition, 30 participants were recruited for this cross-sectional study to increase sample size in the older age group (≥80 y) in order to balance age distribution in the whole sample. We aimed to approximate the proportion of older adults living in senior residences in our sample with the Swiss population average [32]. Data collection was performed at Geriatrische Klinik St.Gallen, Switzerland. The study protocol was approved by the local ethics committee of the canton St.Gallen, Switzerland (study-number: EKSG 14/005, SNCTP 000000039) and the initial training intervention trial was registered at Current Controlled Trials ISRCTN70130279. Our reporting adheres to the STROBE recommendations on what should be included in an accurate and complete report of an observational cross-sectional study [33].

The participants that enrolled to the aforementioned training intervention trial were recruited in August and September 2012 through a newspaper article, a local seniors organization [34], senior residence facilities, primary care physicians, and via the websites of the city’s geriatric hospital [35] and the department of sports of the canton St.Gallen [36]; testing sessions succeeded in October 2012. The additional 30 participants were recruited in February and March 2014 among patients receiving ambulatory or inpatient physical therapy at the city’s geriatric hospital and were tested in the same period. For eligibility, participants had to be older than 70 years, live independently or at senior residence facilities, and sign informed consent. Participants had to be able to walk at least 20 meters, with or without walking aids, for gait analysis. Older adults with Alzheimer’s disease, other type of dementia, or recent head injury were excluded. Judgment by their primary care physician was required in the case of acute or instable chronic diseases (e.g. stroke, diabetes) and rapidly progressing or terminal illnesses before accepting a person for participation.

### Measurements

#### Walking speed

In the 90 participants that were also enrolled for the training intervention trial, walking speed (among other spatio-temporal gait parameters) was assessed at baseline using the GAITRite electronic walkway system (CIR Systems, Havertown, USA) with the Platinum Version 4.0 software. The validity and reliability of the GAITRite system has been well established [37–39]. Walking was initiated two meters before the 7.3-meter active area of the walkway and ended 2 meters thereafter to allow for steady state gait assessment [40].

In the group of 30 participants that were recruited additionally for this study, walking speed was assessed using photo-electric barriers (Brower Timing Systems, Draper, USA) since other gait parameters were not required. The two photo-electric barriers were set up at a height of 50 cm and measured steady state walking time over 8 meters distance. Therefore, participants started walking 2 meters before the first photo-electric barrier and were asked to walk steadily until the finish line 2 meters after the second photo-electric barrier. The difference in walking distance (7.3 vs. 8 meters) for speed assessment in the two groups of participants described above is not expected to be a confound, since previous research has shown a high level of validity and comparability for walking speed assessment at differing distances between 5 and 10 meters in older adults [40–42].

The testing protocol comprised single- and dual-task walking, in which subjects were instructed to walk under four different conditions: at self-selected preferred walking speed, at a fast walking speed (as fast as possible without running), and each with or without a concurrent cognitive task. Each test condition was repeated three times and the mean value was used for statistical analyses. With participants deemed to be less resilient by the investigator, only two repetitions per walking condition were performed to prevent bias from potential fatigue [43]. Two repetitions per walking condition still represent high measurement reliability for walking speed assessments [39, 44]. Trials were repeated when a participant stopped walking or performing the cognitive task. The cognitive–motor dual-task condition was adjusted to the participant’s cognitive abilities to ensure a similar relative cognitive load among all participants. This approach is supported by a meta-analysis that advised to fit the complexity of the cognitive dual-task to the capacity of the target population [45]. The three levels of cognitive task difficulty that were added to the walking task consisted of either a) counting backwards in steps of seven from a random number between 200 and 250, or b) the same calculation task with steps of three, or c) enumerating objects (e.g. flowers, country names, or first names). The purpose of this procedure was to quantify cognitive–motor interference while walking [46]. In order to define the appropriate level of cognitive task difficulty, the participants were first asked to perform the most difficult task (counting backwards in steps of seven) in a standing position without walking. If they were able to accomplish this calculation task in a continuous manner, it was chosen to be performed during the walking assessments. Otherwise, the next easier task (counting backwards in steps of three) was tested without walking, etc. Meta-analytic data showed no differential effects on walking speed among cognitive tasks involving internal interfering factors, such as the mental tracking and verbal fluency tasks applied in the present study [46]. These tasks may also share complex neural networks [47, 48], which interfere with those of gait control [46]. Participants were instructed not to prioritize either task and were allowed to use assistive walking devices. Relative dual-task costs (DTC) were calculated as percentage of loss relative to the single-task walking performance, according to the formula DTC (%) = 100 × (dual-task score − single-task score) ∕ single-task score [25].

#### Secondary outcomes

To assess the association between walking speed and functional lower extremity strength a *5-chair-rises test* was performed in accordance with the criteria described for the Short Physical Performance Battery [49]. Full criteria for test administration are available at the National Institute on Aging website [50]. In addition, the following data were registered: *date of birth*, *sex*, *educational level* (including four options: secondary school = 9 years, apprenticeship = 13 years, grammar school = 13 years, university/higher education = 17 years), *habitation* (including four options: independent, apartment for older adults, senior residence, special-care home), *subjective health rating* (including four options: very good, good, medium, poor), *retrospective fall frequency* for the 6-month period prior to the study (including three options: no falls, one fall, more than one fall; a fall was defined as “an event which results in a person coming to rest inadvertently on the ground or floor or other lower level” [51]), and *walking aids* (including three options: no walking aid, cane, walking frame).

### Statistical analyses

Group differences in the socio-demographic measures of age and education years were compared with student’s independent *t*-tests. Intra-individual differences between walking conditions were analyzed using student’s paired *t*-tests. Inter-individual differences within walking conditions between age groups and sexes were analyzed using student’s independent *t*-tests. Pearson’s correlation for parametric data or Spearman’s correlation and Kendall’s τ for non-parametric data, were applied to analyze the relationships between walking speed and other parameters. Missing data were excluded from the analysis and are specified in the results section. Statistical calculations were performed with IBM SPSS Statistics software for Macintosh, version 23.0 (IBM Corp., Armonk, NY) with a significance level of *α* = .05. Effect size *r* from *t*-tests, was defined as small at *r* = .10, medium at *r* = .30, and large at *r* = .50 and above [52].

## Results

Table 1 shows socio-demographic characteristics of the two age groups. The age ranges in the group ≥80 years were 80–96 years for women and 80–94 years for men, respectively. In each of the following variables one value was missing: *education level* (women 70–79 y), *subjective health* (men 70–79 y), and *walking aids* (women ≥80 y). The complete dataset on which this publication is based, is provided in the supporting information file S1 Dataset.

**Table 1:** Socio-demographic characteristics of the two age groups.

	70–79 years		≥80 years	
	Women	Men	Women	Men
***N***, *n* (%)	34 (57.6%)	25 (42.4%)	44 (72.1%)	17 (27.9%)
**Age** (years), mean ± SD	74.3 ± 0.5	75.5 ± 0.5	85.0 ± 3.8^**t**^	86.8 ± 4.0
**Habitation**, *n* (%)				
* independent*	32 (94.1%)	25 (100%)	33 (75.0%)	12 (70.6%)
* apartment for older adults*	1 (2.9%)	0	2 (4.5%)	1 (5.9%)
* senior residence*	1 (2.9%)	0	9 (20.5%)	4 (23.5%)
* special-care home*	0	0	0	0
**Education** (years), mean ± SD	13.0 ± 0.4	13.6 ± 0.4	12.5 ± 2.0*	13.9 ± 2.7
**Subjective health**, *n* (%)				
* very good*	4 (11.8%)	4 (16.0%)	3 (6.8%)	1 (5.9%)
* good*	18 (52.9%)	11 (44.0%)	19 (43.2%)	10 (58.8%)
* medium*	12 (35.3%)	7 (28.0%)	22 (50.0%)	5 (29.4%)
* poor*	0	2 (8.0%)	0	1 (5.9%)
**Falls last 6 months**, *n* (%)				
* 0*	19 (55.9%)	19 (76.0%)	28 (63.6%)	11 (64.7%)
* 1*	11 (32.4%)	5 (20.0%)	13 (29.5%)	4 (23.5%)
* >1*	4 (11.8%)	1 (4.0%)	3 (6.8%)	2 (11.8%)
**Walking aids**, *n* (%)				
* none*	27 (79.4%)	23 (92.0%)	27 (61.4%)	9 (52.9%)
* cane*	5 (14.7%)	0	9 (20.5%)	5 (29.4%)
* walking frame*	2 (5.9%)	2 (8%)	7 (15.9%)	3 (17.6%)

SD, standard deviation.

^**t**^ = trend for statistical difference between sexes within age group (*p* = .097).

* = significant statistical difference between sexes within age group (*p* = .023).

### Walking speed

**Fig 1:**
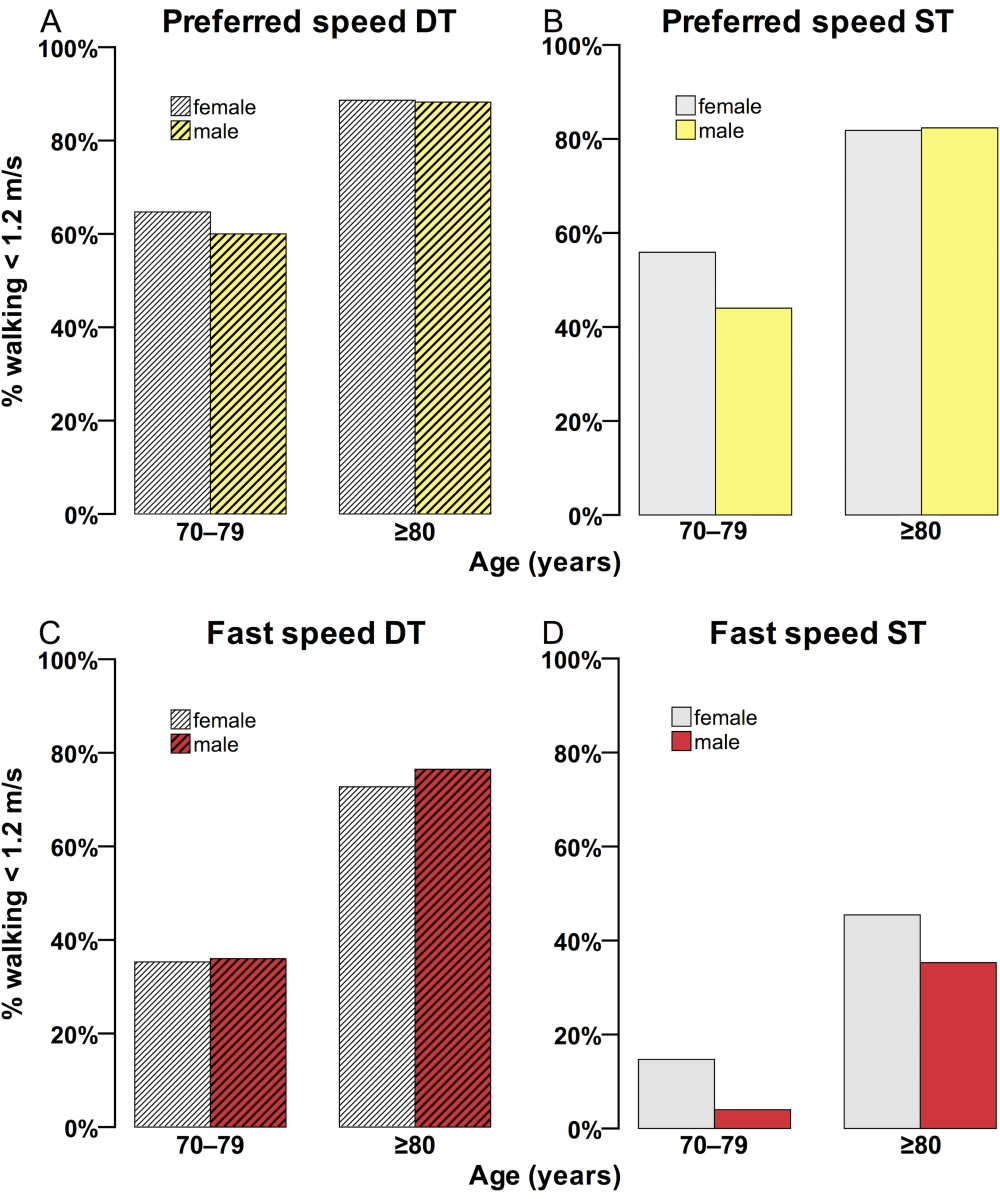
Percentage of women and men walking slower than 1.2 m/s. The graphs represent each of the four walking conditions (A–D). DT, dual-task; ST, single-task.

Fig 1 illustrates the percentage of women and men that were walking slower than 1.2 m/s under the four walking conditions. Differences in walking speed between sexes were not statistically significant. The highest percentage of participants, comprising women and men, walking slower than 1.2 m/s was evident in the *preferred speed dual-task (DT)* walking condition (age group 70–79 years: 62.7%, age group ≥80 years: 88.5%), followed by *preferred speed single-task (ST)* walking (70–79 y: 50.8%, ≥80 y: 82.0%), *fast speed DT* walking (70–79 y: 35.6%, ≥80 y: 73.8%), and *fast speed ST* walking (70–79 y: 10.2%, ≥80 y: 42.6%).

**Fig 2:**
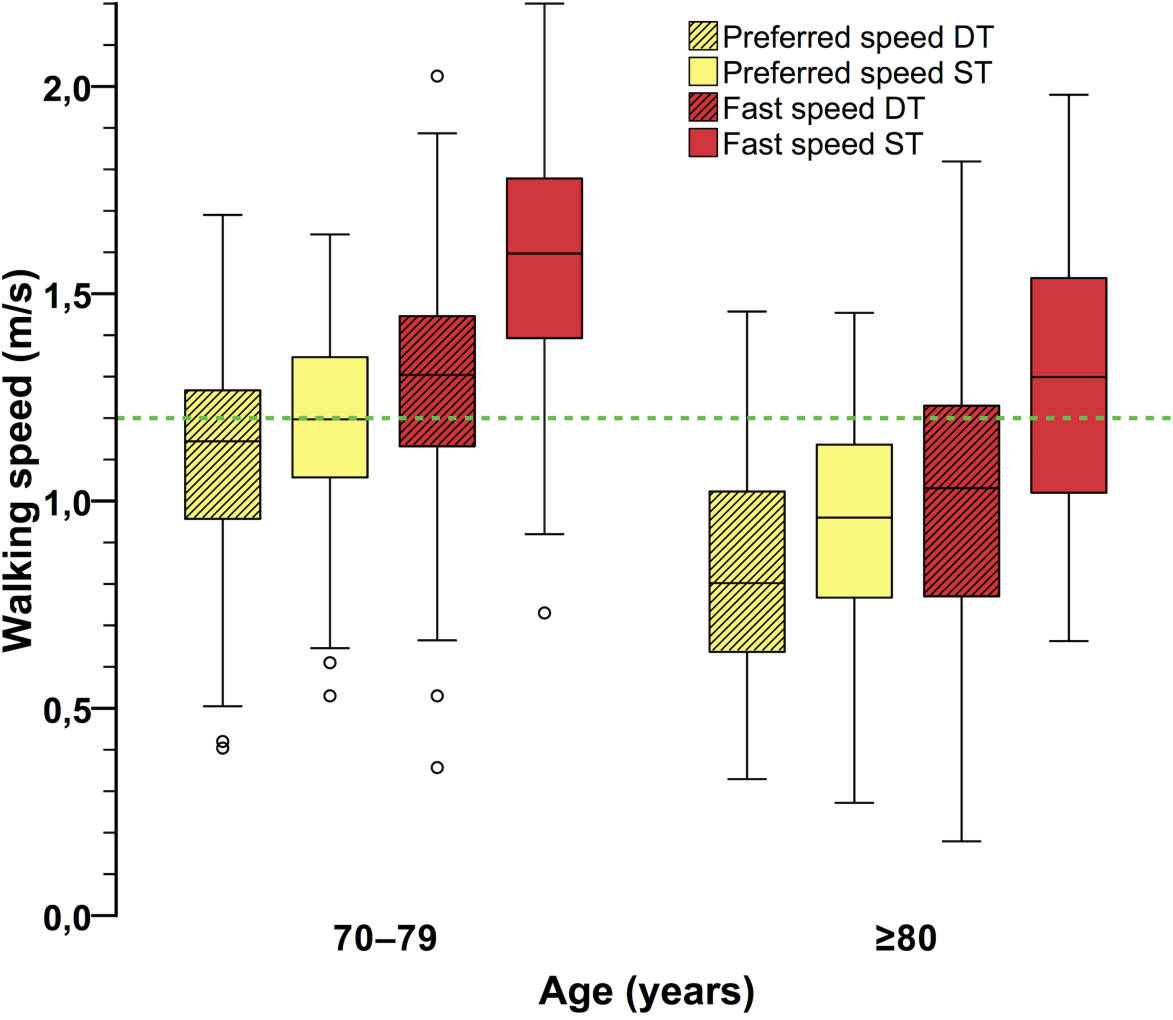
Walking speed under the four walking conditions. Mean walking speed was higher in the younger age group in all four respective walking conditions (all *p* < .001). Mean values of adjacent boxplots in the respective age group are significantly different (all *p* < .05). The dashed green line represents the required 1.2 m/s walking speed to cross streets safely within the green–yellow phase of pedestrian lights. DT, dual-task; ST, single-task.

Fig 2 shows the boxplots for the walking speed measurements under the four walking conditions, separated by age group. The younger participants were walking significantly faster under all respective walking conditions (all *p* < .001, *r* range from .41 to .43). Walking speed was significantly reduced in the DT walking conditions compared to ST walking (age group 70–79 years, *preferred speed DT* vs. *ST*: *t*(58) = −3.34, *p* = .001, *r* = .40; *fast speed DT* vs. *ST*: *t*(58) = −8.86, *p* < .001, *r* = .76; age group ≥80 years, *preferred speed DT* vs. *ST*: *t*(60) = −4.26, *p* < .001, *r* = .48; *fast speed DT* vs. *ST*: *t*(60) = −12.35, *p* < .001, *r* = .85). Additionally, walking speed was significantly lower in *preferred ST* walking than in *fast DT* walking (70–79 y: *t*(58) = −3.09, *p* = .003, *r* = .38; ≥80 y: *t*(60) = −2.50, *p* = .015, *r* = .31). No missing values were present for any walking speed measures.

*Dual-task costs (DTC)* were not different between age groups for preferred speed walking (*p* = .624), whereas for fast speed walking a trend for significantly smaller DTC in the younger group existed (*t*(118) = 1.84, *p* = .069, *r* = .17). Fast speed DTC were significantly higher than preferred speed DTC in both age groups (70–79 y: *t*(58) = 7.68, *p* < .001, *r* = .71; ≥80 y: *t*(60) = 4.38, *p* < .001, *r* = .49). No differences in DTC between sexes were found. Performance data for all four walking conditions, as well as DTC, are summarized in Table 2.

**Table 2:** Walking speed, dual-task costs, and 5-chair-rises-test performance.

	70–79 years		≥80 years	
	Women	Men	Women	Men
**Walking speed** (m/s), mean ± SE				
* Preferred DT*	1.08 ± 0.05	1.13 ± 0.05	0.83 ± 0.04	0.86 ± 0.06
* Preferred ST*	1.17 ± 0.04	1.21 ± 0.05	0.94 ± 0.04	0.96 ± 0.06
* Fast DT*	1.25 ± 0.05	1.33 ± 0.06	0.99 ± 0.05	1.03 ± 0.06
* Fast ST*	1.52 ± 0.05	1.63 ± 0.06	1.28 ± 0.05	1.31 ± 0.08
**Dual-task costs** (%), mean ± SE				
* Preferred*	−7.3 ± 3.2%	−6.3 ± 2.4%	−8.0 ± 4.0%	−10.4 ± 3.6%
* Fast*	−17.0 ± 2.5%	−18.2 ± 2.2%	−22.8 ± 2.3%	−20.6 ± 3.0%
**5-chair-rises test** (s), mean ± SE	11.2 ± 0.5	10.4 ± 0.7	12.9 ± 0.7	11.9 ± 0.9

SE, standard error; DT, dual-task; ST, single-task.

### Correlation with the 5-chair-rises test

**Fig 3:**
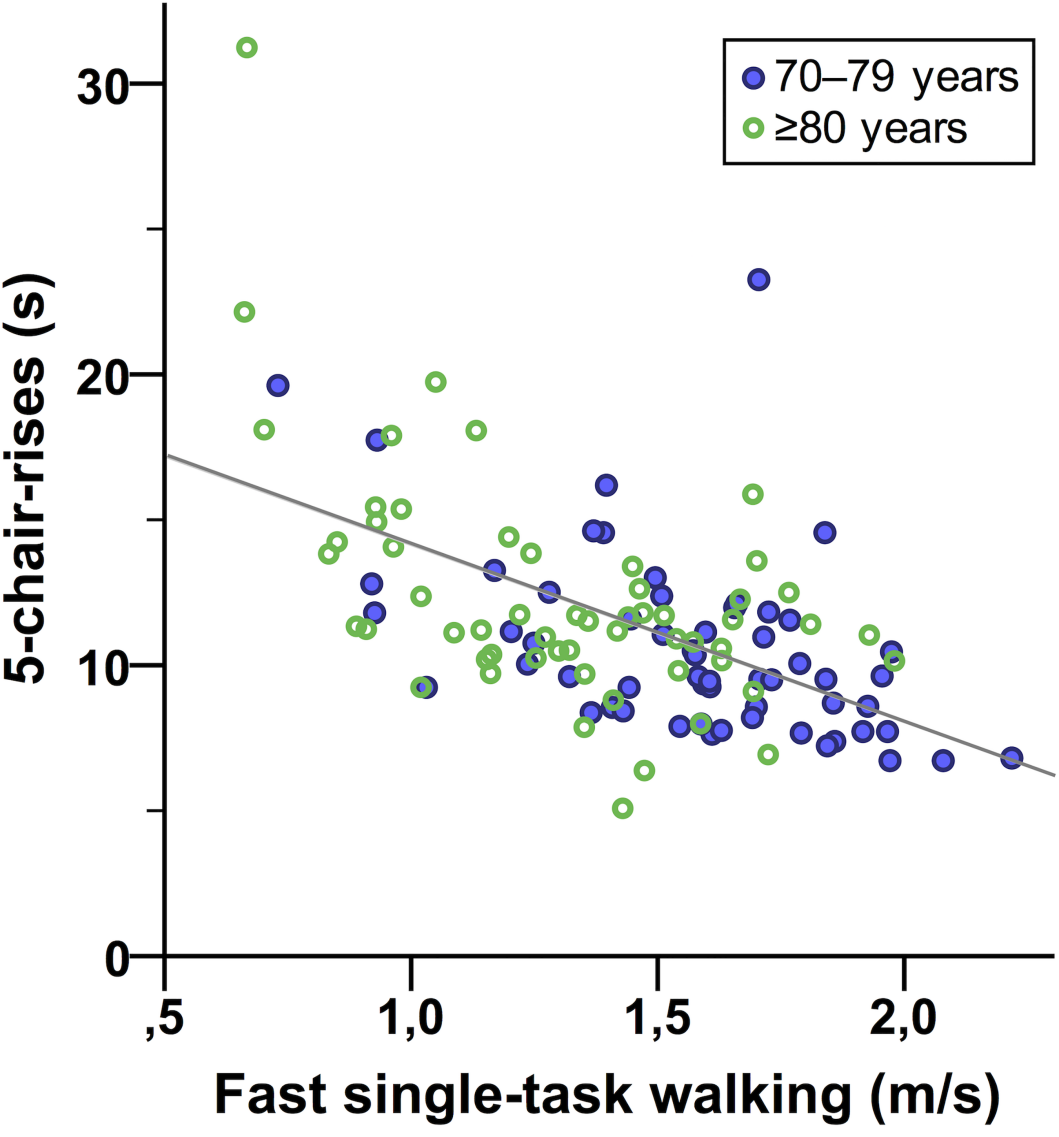
Association between fast single-task walking speed and performance in the 5-chair-rises test. The gray line represents the regression line (y = 20.53 − 6.09 × x, R^2^ linear = 0.310).

A significant association was found between better performance (= shorter time duration) in the *5-chair-rises test* and higher preferred and fast ST walking speeds, respectively (*r*_*s*_ = −.49, 95% BCa CI [−.628, −.350], *p* < .001; *r*_*s*_ = −.51, 95% BCa CI [−.636, −.355], *p* < .001, Fig 3). No difference in 5-chair-rises test performance was evident between sexes within the age groups. The younger age group was significantly faster at completing the 5-chair-rises test (*t*(113) = −2.54, *p* = .012, *r* = .23). Missing values in the 5-chair-rises test were present for one male participant in age group 70–79 years, and for two female and two male participants in age group >80 years. Performance data for the 5-chair-rises test are presented in Table 2.

### Correlation with subjective health and fall frequency

Better *subjective health ratings* were significantly related to higher preferred and fast ST walking speeds, respectively, (τ = .27, 95% BCa CI [.134, .404], *p* < .001; τ = .29, 95% BCa CI [.143, .425], *p* < .001). The same result was found for the relation with the 5-chair-rises test (τ = −.22, 95% BCa CI [.072, .352], *p* = .003). Lower *retrospective fall frequency* was significantly associated with higher preferred and fast ST walking speeds, and shorter 5-chair-rises test time, respectively (τ = −.23, 95% BCa CI [−.360, −.096], *p* = .002; τ = −.20, 95% BCa CI [−.334, −.053], *p* = .009; τ = .27, 95% BCa CI [.123, .414], *p* < .001). Data for subjective health ratings and retrospective fall frequency are presented in Table 1.

## Discussion

This study aimed to identify in a convenience sample of Swiss older adults the proportion that doesn’t reach 1.2 m/s walking speed, which is required to safely cross streets within the green–yellow phase of pedestrian lights, while walking fast under a cognitively challenging dual-task condition. Our main finding is that 35.6% of Swiss older adults of our convenience sample in the age group of 70–79 years and 73.8% in the age group older than 80 years are not able to walk faster than 1.2 m/s under fast speed dual-task walking. Therefore, it seems feasible to conclude that a similar proportion of older adults is not capable of crossing streets safely within the green–yellow phase of pedestrian lights. These results, first, suggest that the fitness status of many older adults seems not to be appropriate to safely encounter the requirements for pedestrians in urban areas and, second, reinforce the need for regular cognitive and physical training in the older population [53] to keep up with the demands of daily life in the community.

Notably, to date no other study that stated a discrepancy between older adults walking ability and the requirements to cross streets at pedestrian lights, has interpreted their findings in the view of older adult’s lack of fitness. In contrast, these researchers recommended that policy makers should think about reducing the walking speed requirements at pedestrian lights [13–17, 19]. However, we would argue that this recommendation is tackling the problem of impaired walking and street crossing ability in older adults only indirectly. Generally, there are strong indications that cognitive and physical factors are related to the observed slowing of walking speed in older adults [12, 53], and recent research demonstrated that aging-related sensorial, cognitive and physical changes, have a major negative impact on older pedestrian’s mobility [10]. Furthermore, walking speed and cognitive measures (e.g. processing speed and spatial planning) are predictive of unsafe street crossing behavior in old adults [54]. Therefore, the most direct way of enhancing older pedestrians’ safety would include training of their cognitive and physical capacities [10, 54]. Particularly, walking speed is modifiable through strength training [55] and other physical exercise modalities [56] and is trainable until old age [55, 57, 58]. Cognitive components are improvable, for instance, through aerobic training [57, 58] or recent combined cognitive–physical training approaches [31, 59]. Consequently, older adults should invest time in modifying cognitive and physical capacities to improve their street crossing behavior, which represents one of the main components of pedestrian activity that older adults should be able to perform in order to maintain independence in daily functioning [10].

### Proportion of older adults walking slower than 1.2 m/s

A wide range of proportions of older adults walking slower than 1.2 m/s have been described previously [13–17, 19], ranging from 31% [13] to over 99% [17]. These proportions varied depending on the heterogeneity of the included samples; e.g. participants’ age and health status, walking test conditions (preferred or fast speed, single- or dual-task), measurement methods (steady-state speed or speed including an acceleration phase), and sample size. Regarding these factors, the study by Donoghue and colleagues, examining an Irish nationally representative sample of 4909 participants, seems best suited for comparisons with our study. They similarly applied steady state walking speed measurements and were the only researchers to date that included a preferred speed dual-task walking condition [13]. Nonetheless, this is the first study applying a *fast speed dual-task walking* condition to approximate the real-life situation of crossing streets at pedestrian lights under cognitive challenges and time pressure. We found that under this test condition the proportion of older adults walking slower than 1.2 m/s is smaller compared to our preferred speed single- and dual-task walking results, thus confirming our hypothesis. In addition, our novel approach resulted in a more conservative estimation of the proportion of older adults that are at risk when crossing streets at pedestrian lights compared to the preferred speed dual-tasks walking approach applied by Donoghue and colleagues [13].

Comparing the *preferred speed dual-task walking* conditions, fewer of our Swiss 70–79 years old group were slower than 1.2 m/s (62.7%) than in the aforementioned Irish 65–74 years old group (76%) [13]. In the older age groups, this difference between the two studies was not present with almost identical values (88.5% Swiss vs. 91% Irish <1.2 m/s) [13]. We hypothesize that the discrepancy in the dual-task condition might have occurred due to disparate cognitive tasks that were added. In our study, a serial subtracting task was applied and adapted to each participant’s abilities. On the other hand, Donoghue et al. asked their participants to recite alternate letters of the alphabet (e.g. A–C–E etc.) [13]. Apparently, in the younger participants, the letters task slowed walking speed more than the subtracting task. This effect might not have come into play in the older participants due to a similarly high percentage of around 90% in both studies. Nevertheless, Al-Yahya and colleagues asserted a lack of evidence regarding differential effects of specific cognitive tasks on walking speed [46]. Based on meta-analytical data, it was concluded that in general, tasks involving internal interference (e.g. mental tracking tasks, such as those described above) would disturb walking performance more than tasks involving external interference (e.g. reaction time tasks) [45, 46].

The younger participants in our study (70–79 y) tended to exhibit lower *fast walking speed dual-task costs* compared to the older participants (≥80 y). This finding might reflect a cognitive advantage, since dual-task effects on walking speed, induced by mental tracking tasks, are strongly related with cognitive status as was shown by meta-analytical evidence [46]. In addition, our result is in line with the notion that younger adults (<40 y) demonstrated consistently less interference on walking speed from concurrent cognitive tasks than healthy older adults [46]. Similarly, Zito and colleagues reported that older adults looked more at the ground while crossing a virtual street and to a lesser extent at the other side of the street compared to younger adults [60]. Together, these cognitive–motor dual-tasking effects could be explained with the *resource-based attentional model* which proposes that the total capacity of attentional resources is limited and declines at older age, causing problems in dual-task situations where attentional resources are shared between competing tasks [61, 62].

In the *preferred speed single-task walking* condition, about 20% more older adults in our sample walked slower than 1.2 m/s in both age groups (70–79 y and ≥80 y) when compared to the Donoghue et al. study. This discrepancy might be explained by the fact that participants in the latter study were divided into groups that were five years younger (65–74 y and ≥75 y) [13]. *Fast speed single-task walking* was assessed by one previous study. Amosun and colleagues tested 47 South African participants aged 65–93 years and reported that 34% walked slower than 1.2 m/s which is between the percentages of our two age groups showing 10.2% and 42.6%, respectively [19].

### Implications for cognitive and physical training

Recently, Leisman and colleagues argued that cognitive and motor processes are functionally connected, providing ample clinical and neural evidence supporting their dynamic bidirectional influences [63]. For instance, early impairments of cognitive processes, including attention, executive function, and working memory, were shown to be associated with slowed single- and dual-task walking speed and instability [64, 65]. The interrelation of cognition and gait in combination with the cognitive–motor dual-task characteristics of crossing streets at pedestrian lights asks for combined cognitive and physical training measures to improve older people’s mobility and safety on public streets [60, 66].

Several studies attained positive effects from physical training on walking speed. For instance, Plummer and colleagues reported significant effects of physical exercise on single- and dual-task walking speed in their meta-analysis. The interventions included varied types of exercise with or without dual-task components. The authors concluded that improvements in dual-task walking were primarily on account of an increase in walking speed under dual-task conditions and not due to reduced dual-task costs [67]. Another recent meta-analysis by Van Abbema and colleagues demonstrated that progressive strength training (at 70–80% of the one repetition maximum) was most effective to improve preferred walking speed, followed by exercise with a rhythmic component, and combined strength, balance, and endurance training [55]. This concurs with our finding that higher walking speed correlated significantly with better functional lower extremity strength as measured with the 5-chair-rises test.

Moreover, in a recent combined cognitive–physical multicomponent training intervention with 71 older adults we have shown a 12% increase in preferred walking speed (1.16 ± 0.24 m/s to 1.30 ± 0.25 m/s from pre- to post-test, respectively; mean ± SD) and a concurrent improvement of 13% in preferred dual-task walking speed (1.05 ± 0.29 m/s to 1.19 ± 0.31 m/s) [25]. Thereby, it seems noteworthy that an increase in preferred walking speed of 0.1 m/s was established as substantial and meaningful [2, 68, 69]. Interestingly, when translating the data of the latter study into the context of crossing streets at pedestrian lights, 42.6% of the participants (mean age 78.9 ± 5.5 years) were not able to walk faster than 1.2 m/s under dual-task conditions at baseline, whereas after 6 months of training this percentage decreased to 25.5% (calculation based on original data from [25]). Additionally, the substantial improvements in walking speed were accompanied by broad improvements in various cognitive domains (including executive functions and attention) [31]. Such training-induced cognitive improvements appear to be mediated by functional brain adaptations in the prefrontal cortex during challenging walking [59]. These adaptions might free up attentional resources that could be potentially useful when crossing streets in addition to improvements in walking speed.

### Walking speed and health-related parameters

Further support for the benefits of an adequate walking speed level and appropriate lower extremity strength is provided by their association with health-related parameters. Our results indicate a significant association between the *subjective health rating* and both, walking speed and functional lower extremity strength (5-chair-rises test), respectively. This finding is in line with other studies reporting a strong relationship between reduced walking speed and adverse health outcomes in older adults [1, 2, 70]. Thereby, preferred walking speed below 0.8 m/s (in a 4-meter walk) was established as a sensible and often used cut-point to identify persons at risk of adverse outcomes [2]. Moreover, our correlation analyses also show significant relations of *retrospective fall frequency* with both, walking speed and functional lower extremity strength. Similarly, a meta-analysis demonstrated that single- and dual-task walking speed tests were equally applicable to predict falls in older people [71]. However, findings appear to be controversial in this context, since a recent systematic review concluded that future fall risk is stronger related to dual-task than single-task gait testing [72].

### Strengths and limitations

A methodological strength of this study is the integration of four different walking conditions, comprising single- and dual-task walking at preferred and fast speed, to approximate real-life street crossing behavior at pedestrian lights. However, some limitations should be considered. First, the applied measurements of gait speed do only reflect an approximation of the conditions at real pedestrian lights, since the measurements were performed indoors in a protected environment, which implies a lack of ecological validity [73]. A second limitation of this study is that it includes a convenience sample of participants that either intended to participate in a prospective training intervention or that were additionally recruited for cross-sectional walking speed assessments. Therefore, it is possibly not representative for the general population of (Swiss) older adults and the data does not reflect normative aging. However, because average preferred single- and dual-task walking speeds in our heterogenic sample were comparable to values reported in two recent meta-analyses [29, 74], we assume that our measurements are reasonably representative. Moreover, the distribution of participants living independently vs. those living in senior residences was approximately in the range of the distribution in the Swiss older population (70–79 y: 1.7% of our sample living in senior residences vs. 1.7–4.8% of Swiss population, respectively; ≥80 y: 21.3% vs. 8.5–30.3%) [32]. Another, third, limitation of the presented data is that actual crosswalk speed requirements do not always follow the 1.2 m/s guideline, but appear to vary widely [15, 19, 75]. A recent systematic review, for instance, reported mean walking speed requirements of 0.44, 0.73–0.78, and 1.32 m/s, in the large cities of Melbourne, Singapore, and Los Angeles, respectively [75]. However, considering the limited number of crossroads that were analyzed in these studies, it still seems adequate to compare older adult’s walking speed with the 1.2 m/s standard recommended by national traffic authorities, including the United States, Canada, the United Kingdom, Ireland, South Africa, and Switzerland [11, 13–15, 18, 19].

## Conclusions

This study demonstrates that about every third (35.6%) older person at the age of 70–79 years and almost three-quarters (73.8%) of persons ≥80 years cannot walk faster than 1.2 m/s, which is required to cross streets safely within the green–yellow phase of pedestrian lights, under cognitively challenging conditions (fast speed dual-task walking). We propose that fast speed dual-task walking is more reflective of the time pressure and cognitive challenge at real pedestrian lights than the previously applied assessments of single- or dual-task walking at preferred speed. This novel approach led to a more conservative estimation of the proportion of older adults that are unable to walk as fast as 1.2 m/s compared to a previous investigation assessing dual-task walking at preferred speed [13]. Nonetheless, this proportion is still alarmingly high, given the fact that slowed walking speed is related to various adverse health outcomes [1, 2, 64, 65].

We therefore strongly recommend acknowledging the results of this and other similar studies as evidence that the fitness status of many older people is inadequate for maintaining their mobility-related independence in daily life and their safety as pedestrians in urban areas. In the long run, it clearly appears more fruitful to promote direct measures that improve the older population’s cognitive and physical fitness instead of tackling the problem indirectly by adapting pedestrian light’s settings as proposed by preceding studies [13–17, 19]. Slow walking speed, as the main cause of the problem, is modifiable particularly through strength and other training modalities [55, 56], while the cognitive, attentional components are improvable through aerobic [57, 58] and other exercise training modes [76, 77], or recent promising combined cognitive–physical training approaches [31, 59]. Future research should, therefore, investigate combinations of cognitive and physical training strategies targeting the dual-task requirements of safe street crossing behavior related to gait speed and assess their effectiveness in ecologically valid settings.

## Supporting information

S1 Dataset Complete dataset on which the present study is based. (XLSX)

## References

[1] Liu B, Hu X, Zhang Q, Fan Y, Li J, Zou R, et al Usual walking speed and all-cause mortality risk in older people: A systematic review and meta-analysis. Gait Posture. 2016;44: 172–7. doi: 10.1016/j.gaitpost.2015.12.008 27004653 10.1016/j.gaitpost.2015.12.008

[2] Abellan van Kan G, Rolland Y, Andrieu S, Bauer J, Beauchet O, Bonnefoy M, et al Gait speed at usual pace as a predictor of adverse outcomes in community-dwelling older people an International Academy on Nutrition and Aging (IANA) Task Force. J Nutr Health Aging. 2009;13(10): 881–9. 19924348 10.1007/s12603-009-0246-z PMC12878092

[3] Fritz S, Lusardi M. White paper: "walking speed: the sixth vital sign". J Geriatr Phys Ther. 2009;32(2): 46–9. 20039582

[4] Studenski S. Bradypedia: is gait speed ready for clinical use? J Nutr Health Aging. 2009;13(10): 878–80. 19924347 10.1007/s12603-009-0245-0 PMC12878062

[5] Turner G, Clegg A, British Geriatrics S, Age UK, Royal College of General P. Best practice guidelines for the management of frailty: a British Geriatrics Society, Age UK and Royal College of General Practitioners report. Age Ageing. 2014;43(6): 744–7. doi: 10.1093/ageing/afu138 25336440 10.1093/ageing/afu138

[6] Peel NM, Kuys SS, Klein K. Gait speed as a measure in geriatric assessment in clinical settings: a systematic review. J Gerontol A Biol Sci Med Sci. 2013;68(1): 39–46. doi: 10.1093/gerona/gls174 22923430 10.1093/gerona/gls174

[7] Chen PJ, Lin MH, Peng LN, Liu CL, Chang CW, Lin YT, et al Predicting cause-specific mortality of older men living in the Veterans home by handgrip strength and walking speed: a 3-year, prospective cohort study in Taiwan. J Am Med Dir Assoc. 2012;13(6): 517–21. doi: 10.1016/j.jamda.2012.02.002 22459909 10.1016/j.jamda.2012.02.002

[8] Dumurgier J, Elbaz A, Ducimetiere P, Tavernier B, Alperovitch A, Tzourio C. Slow walking speed and cardiovascular death in well functioning older adults: prospective cohort study. BMJ. 2009;339: b4460 doi: 10.1136/bmj.b4460 19903980 10.1136/bmj.b4460 PMC2776130

[9] Studenski S, Perera S, Wallace D, Chandler JM, Duncan PW, Rooney E, et al Physical performance measures in the clinical setting. J Am Geriatr Soc. 2003;51(3): 314–22. 12588574 10.1046/j.1532-5415.2003.51104.x

[10] Tournier I, Dommes A, Cavallo V. Review of safety and mobility issues among older pedestrians. Accid Anal Prev. 2016;91: 24–35. doi: 10.1016/j.aap.2016.02.031 26950033 10.1016/j.aap.2016.02.031

[11] Brown KC, Hanson HM, Firmani F, Liu D, McAllister MM, Merali K, et al Gait Speed and Variability for Usual Pace and Pedestrian Crossing Conditions in Older Adults Using the GAITRite Walkway. Gerontol Geriatr Med. 2015;1: 2333721415618858 doi: 10.1177/2333721415618858 28138480 10.1177/2333721415618858 PMC5119883

[12] Busch TD, Duarte YA, Pires Nunes D, Lebrao ML, Satya Naslavsky M, dos Santos Rodrigues A, et al Factors associated with lower gait speed among the elderly living in a developing country: a cross-sectional population-based study. BMC Geriatr. 2015;15: 35 doi: 10.1186/s12877-015-0031-2 25880124 10.1186/s12877-015-0031-2 PMC4391309

[13] Donoghue OA, Dooley C, Kenny RA. Usual and Dual-Task Walking Speed: Implications for Pedestrians Crossing the Road. J Aging Health. 2016;28(5): 850–62. doi: 10.1177/0898264315614004 26578545 10.1177/0898264315614004

[14] Asher L, Aresu M, Falaschetti E, Mindell J. Most older pedestrians are unable to cross the road in time: a cross-sectional study. Age Ageing. 2012;41(5): 690–4. doi: 10.1093/ageing/afs076 22695790 10.1093/ageing/afs076

[15] Bollard E, Fleming H. A study to investigate the walking speed of elderly adults with relation to pedestrian crossings. Physiother Theory Pract. 2013;29(2): 142–9. doi: 10.3109/09593985.2012.703760 22844989 10.3109/09593985.2012.703760

[16] Romero-Ortuno R, Cogan L, Cunningham CU, Kenny RA. Do older pedestrians have enough time to cross roads in Dublin? A critique of the Traffic Management Guidelines based on clinical research findings. Age Ageing. 2010;39(1): 80–6. doi: 10.1093/ageing/afp206 19923163 10.1093/ageing/afp206

[17] Langlois JA, Keyl PM, Guralnik JM, Foley DJ, Marottoli RA, Wallace RB. Characteristics of older pedestrians who have difficulty crossing the street. Am J Public Health. 1997;87(3): 393–7. 9096539 10.2105/ajph.87.3.393 PMC1381010

[18] Lichtsignalanlagen: Übergangszeiten und Mindestzeiten. Vereinigung Schweizerischer Strassenfachleute; 1992. Available from: http://www.vss.ch/de/webviewer/viewdocument/4096/dHash/e93858adbefa1adbf71b2899ba382451a7133eb2/.

[19] Amosun SL, Burgess T, Groeneveldt L, Hodgson T. Are elderly pedestrians allowed enough time at pedestrian crossings in Cape Town, South Africa? Physiother Theory Pract. 2007;23(6): 325–32. doi: 10.1080/09593980701593755 18075906 10.1080/09593980701593755

[20] Fried LP, Tangen CM, Walston J, Newman AB, Hirsch C, Gottdiener J, et al Frailty in older adults: evidence for a phenotype. J Gerontol A Biol Sci Med Sci. 2001;56(3): M146–56. 11253156 10.1093/gerona/56.3.m146

[21] Mirelman A, Herman T, Brozgol M, Dorfman M, Sprecher E, Schweiger A, et al Executive function and falls in older adults: new findings from a five-year prospective study link fall risk to cognition. PLoS One. 2012;7(6): e40297 doi: 10.1371/journal.pone.0040297 22768271 10.1371/journal.pone.0040297 PMC3386974

[22] Herman T, Mirelman A, Giladi N, Schweiger A, Hausdorff JM. Executive control deficits as a prodrome to falls in healthy older adults: a prospective study linking thinking, walking, and falling. J Gerontol A Biol Sci Med Sci. 2010;65(10): 1086–92. doi: 10.1093/gerona/glq077 20484336 10.1093/gerona/glq077 PMC2949331

[23] Nagamatsu LS, Voss M, Neider MB, Gaspar JG, Handy TC, Kramer AF, et al Increased cognitive load leads to impaired mobility decisions in seniors at risk for falls. Psychol Aging. 2011;26(2): 253–9. doi: 10.1037/a0022929 21463063 10.1037/a0022929 PMC3123036

[24] Neider MB, Gaspar JG, McCarley JS, Crowell JA, Kaczmarski H, Kramer AF. Walking and talking: dual-task effects on street crossing behavior in older adults. Psychol Aging. 2011;26(2): 260–8. doi: 10.1037/a0021566 21401262 10.1037/a0021566 PMC3699858

[25] Eggenberger P, Theill N, Holenstein S, Schumacher V, de Bruin ED. Multicomponent physical exercise with simultaneous cognitive training to enhance dual-task walking of older adults: a secondary analysis of a 6-month randomized controlled trial with 1-year follow-up. Clin Interv Aging. 2015;10: 1711–32. doi: 10.2147/CIA.S91997 26604719 10.2147/CIA.S91997 PMC4631411

[26] de Bruin ED, Schmidt A. Walking behaviour of healthy elderly: attention should be paid. Behav Brain Funct. 2010;6: 59 doi: 10.1186/1744-9081-6-59 20939911 10.1186/1744-9081-6-59 PMC2959004

[27] Butler AA, Lord SR, Fitzpatrick RC. Perceptions of Speed and Risk: Experimental Studies of Road Crossing by Older People. PLoS One. 2016;11(4): e0152617 doi: 10.1371/journal.pone.0152617 27054918 10.1371/journal.pone.0152617 PMC4824509

[28] Dommes A, Cavallo V, Oxley J. Functional declines as predictors of risky street-crossing decisions in older pedestrians. Accid Anal Prev. 2013;59: 135–43. doi: 10.1016/j.aap.2013.05.017 23792612 10.1016/j.aap.2013.05.017

[29] Smith E, Cusack T, Blake C. The effect of a dual task on gait speed in community dwelling older adults: A systematic review and meta-analysis. Gait Posture. 2016;44: 250–8. doi: 10.1016/j.gaitpost.2015.12.017 27004667 10.1016/j.gaitpost.2015.12.017

[30] Location, figures and facts. City of St.Gallen, Fachstelle für Statistik Kanton St.Gallen; 2016. Available from: http://www.stadt.sg.ch/home/welcome/st-gallen/geografische-lage-zahlen-fakten.html.

[31] Eggenberger P, Schumacher V, Angst M, Theill N, de Bruin ED. Does multicomponent physical exercise with simultaneous cognitive training boost cognitive performance in older adults? A 6-month randomized controlled trial with a 1-year follow-up. Clin Interv Aging. 2015;10: 1335–49. doi: 10.2147/CIA.S87732 26316729 10.2147/CIA.S87732 PMC4544626

[32] Statistik Alters- und Pflegeinstitutionen. CURAVIVA Schweiz; 2014. Available from: https://www.curaviva.ch/Verlag/PDgwT/?id=4F1159DA-C917-18D4-9E2E6B94512ABB65&method=objectdata.detail&p=3&callerid=&c=DE609894-EC42-78FB-ABEE19D5B63B1482.

[33] von Elm E, Altman DG, Egger M, Pocock SJ, Gotzsche PC, Vandenbroucke JP, et al The Strengthening the Reporting of Observational Studies in Epidemiology (STROBE) statement: guidelines for reporting observational studies. PLoS Med. 2007;4(10): e296 doi: 10.1371/journal.pmed.0040296 17941714 10.1371/journal.pmed.0040296 PMC2020495

[34] Pro Senectute St.Gallen. Available from: http://www.sg.pro-senectute.ch/

[35] Geriatrische Klinik St.Gallen. Available from: http://www.gesundheitundalter.ch/Home/GeriatrischeKlinik/Portrait/tabid/106/Default.aspx.

[36] Amt für Sport Kanton St.Gallen. Available from: http://www.sg.ch/home/bildung/sport/Erwachsene.html.

[37] Webster KE, Wittwer JE, Feller JA. Validity of the GAITRite walkway system for the measurement of averaged and individual step parameters of gait. Gait Posture. 2005;22(4): 317–21. doi: 10.1016/j.gaitpost.2004.10.005 16274913 10.1016/j.gaitpost.2004.10.005

[38] van Uden CJ, Besser MP. Test-retest reliability of temporal and spatial gait characteristics measured with an instrumented walkway system (GAITRite). BMC Musculoskelet Disord. 2004;5: 13 doi: 10.1186/1471-2474-5-13 15147583 10.1186/1471-2474-5-13 PMC420245

[39] Bilney B, Morris M, Webster K. Concurrent related validity of the GAITRite walkway system for quantification of the spatial and temporal parameters of gait. Gait Posture. 2003;17(1): 68–74. 12535728 10.1016/s0966-6362(02)00053-x

[40] Ng SS, Ng PC, Lee CY, Ng ES, Tong MH, Fong SS, et al Assessing the walking speed of older adults: the influence of walkway length. Am J Phys Med Rehabil. 2013;92(9): 776–80. doi: 10.1097/PHM.0b013e31828769d0 23478456 10.1097/PHM.0b013e31828769d0

[41] Kim HJ, Park I, Lee HJ, Lee O. The reliability and validity of gait speed with different walking pace and distances against general health, physical function, and chronic disease in aged adults. J Exerc Nutrition Biochem. 2016;20(3): 46–50. doi: 10.20463/jenb.2016.09.20.3.7 27757387 10.20463/jenb.2016.09.20.3.7 PMC5067420

[42] Middleton A, Fritz SL, Lusardi M. Walking speed: the functional vital sign. J Aging Phys Act. 2015;23(2): 314–22. doi: 10.1123/japa.2013-0236 24812254 10.1123/japa.2013-0236 PMC4254896

[43] van het Reve E, de Bruin ED. Strength-balance supplemented with computerized cognitive training to improve dual task gait and divided attention in older adults: a multicenter randomized-controlled trial. BMC Geriatr. 2014;14: 134 doi: 10.1186/1471-2318-14-134 25511081 10.1186/1471-2318-14-134 PMC4293005

[44] Goldberg A, Schepens S. Measurement error and minimum detectable change in 4-meter gait speed in older adults. Aging Clin Exp Res. 2011;23(5–6): 406–12. 22526072 10.1007/BF03325236

[45] Chu YH, Tang PF, Peng YC, Chen HY. Meta-analysis of type and complexity of a secondary task during walking on the prediction of elderly falls. Geriatr Gerontol Int. 2013;13(2): 289–97. doi: 10.1111/j.1447-0594.2012.00893.x 22694365 10.1111/j.1447-0594.2012.00893.x

[46] Al-Yahya E, Dawes H, Smith L, Dennis A, Howells K, Cockburn J. Cognitive motor interference while walking: a systematic review and meta-analysis. Neurosci Biobehav Rev. 2011;35(3): 715–28. doi: 10.1016/j.neubiorev.2010.08.008 20833198 10.1016/j.neubiorev.2010.08.008

[47] Fuster JM. Cortex and Mind: Unifying Cognition New York: Oxford University Press; 2003.

[48] Gazzaley A, D’Esposito M. Neural Networks: an empirical neuroscience approach toward understanding cognition. Cortex. 2006;42: 1037–40.

[49] Guralnik JM, Simonsick EM, Ferrucci L, Glynn RJ, Berkman LF, Blazer DG, et al A short physical performance battery assessing lower extremity function: association with self-reported disability and prediction of mortality and nursing home admission. J Gerontol. 1994;49(2): M85–94. 8126356 10.1093/geronj/49.2.m85

[50] Guralnik JM. Assessing physical performance in the older patient. 2013. Available from: https://www.irp.nia.nih.gov/branches/leps/sppb/index.htm.

[51] Falls, fact sheet. World Health Organization; 2016. Available from: http://www.who.int/mediacentre/factsheets/fs344/en/.

[52] Cohen J. Statistical power analysis for the behavioral sciences. 2nd ed. Hillsdale, New Jersey: Lawrence Erlbaum; 1988.

[53] Kuh D, Karunananthan S, Bergman H, Cooper R. A life-course approach to healthy ageing: maintaining physical capability. Proc Nutr Soc. 2014;73(2): 237–48. doi: 10.1017/S0029665113003923 24456831 10.1017/S0029665113003923 PMC3981474

[54] Geraghty J, Holland C, Rochelle K. Examining links between cognitive markers, movement initiation and change, and pedestrian safety in older adults. Accid Anal Prev. 2016;89: 151–9. doi: 10.1016/j.aap.2015.12.019 26871616 10.1016/j.aap.2015.12.019

[55] Van Abbema R, De Greef M, Craje C, Krijnen W, Hobbelen H, Van Der Schans C. What type, or combination of exercise can improve preferred gait speed in older adults? A meta-analysis. BMC Geriatr. 2015;15: 72 doi: 10.1186/s12877-015-0061-9 26126532 10.1186/s12877-015-0061-9 PMC4488060

[56] Hortobagyi T, Lesinski M, Gabler M, VanSwearingen JM, Malatesta D, Granacher U. Effects of Three Types of Exercise Interventions on Healthy Old Adults' Gait Speed: A Systematic Review and Meta-Analysis. Sports Med. 2015;45(12): 1627–43. doi: 10.1007/s40279-015-0371-2 26286449 10.1007/s40279-015-0371-2 PMC4656792

[57] Colcombe S, Kramer AF. Fitness effects on the cognitive function of older adults: a meta-analytic study. Psychol Sci. 2003;14(2): 125–30. doi: 10.1111/1467-9280.t01-1-01430 12661673 10.1111/1467-9280.t01-1-01430

[58] Smith PJ, Blumenthal JA, Hoffman BM, Cooper H, Strauman TA, Welsh-Bohmer K, et al Aerobic exercise and neurocognitive performance: a meta-analytic review of randomized controlled trials. Psychosom Med. 2010;72(3): 239–52. doi: 10.1097/PSY.0b013e3181d14633 20223924 10.1097/PSY.0b013e3181d14633 PMC2897704

[59] Eggenberger P, Wolf M, Schumann M, de Bruin ED. Exergame and Balance Training Modulate Prefrontal Brain Activity during Walking and Enhance Executive Function in Older Adults. Front Aging Neurosci. 2016;8: 66 doi: 10.3389/fnagi.2016.00066 27148041 10.3389/fnagi.2016.00066 PMC4828439

[60] Zito GA, Cazzoli D, Scheffler L, Jager M, Muri RM, Mosimann UP, et al Street crossing behavior in younger and older pedestrians: an eye- and head-tracking study. BMC Geriatr. 2015;15: 176 doi: 10.1186/s12877-015-0175-0 26714495 10.1186/s12877-015-0175-0 PMC4696098

[61] Woollacott M, Shumway-Cook A. Attention and the control of posture and gait: a review of an emerging area of research. Gait Posture. 2002;16(1): 1–14. 12127181 10.1016/s0966-6362(01)00156-4

[62] Beurskens R, Bock O. Age-related deficits of dual-task walking: a review. Neural Plast. 2012;2012: 131608 doi: 10.1155/2012/131608 22848845 10.1155/2012/131608 PMC3403123

[63] Leisman G, Moustafa AA, Shafir T. Thinking, Walking, Talking: Integratory Motor and Cognitive Brain Function. Front Public Health. 2016;4: 94 doi: 10.3389/fpubh.2016.00094 27252937 10.3389/fpubh.2016.00094 PMC4879139

[64] Montero-Odasso M, Verghese J, Beauchet O, Hausdorff JM. Gait and cognition: a complementary approach to understanding brain function and the risk of falling. J Am Geriatr Soc. 2012;60(11): 2127–36. doi: 10.1111/j.1532-5415.2012.04209.x 23110433 10.1111/j.1532-5415.2012.04209.x PMC3498517

[65] Gonzales JU, James CR, Yang HS, Jensen D, Atkins L, Thompson BJ, et al Different cognitive functions discriminate gait performance in younger and older women: A pilot study. Gait Posture. 2016;50: 89–95. doi: 10.1016/j.gaitpost.2016.08.021 27585184 10.1016/j.gaitpost.2016.08.021

[66] Forte R, Boreham CA, De Vito G, Pesce C. Health and Quality of Life Perception in Older Adults: The Joint Role of Cognitive Efficiency and Functional Mobility. Int J Environ Res Public Health. 2015;12(9): 11328–44. doi: 10.3390/ijerph120911328 26378556 10.3390/ijerph120911328 PMC4586678

[67] Plummer P, Zukowski LA, Giuliani C, Hall AM, Zurakowski D. Effects of Physical Exercise Interventions on Gait-Related Dual-Task Interference in Older Adults: A Systematic Review and Meta-Analysis. Gerontology. 2015 doi: 10.1159/000371577 25721432 10.1159/000371577

[68] Perera S, Mody SH, Woodman RC, Studenski SA. Meaningful change and responsiveness in common physical performance measures in older adults. J Am Geriatr Soc. 2006;54(5): 743–9. doi: 10.1111/j.1532-5415.2006.00701.x 16696738 10.1111/j.1532-5415.2006.00701.x

[69] Kwon S, Perera S, Pahor M, Katula JA, King AC, Groessl EJ, et al What is a meaningful change in physical performance? Findings from a clinical trial in older adults (the LIFE-P study). J Nutr Health Aging. 2009;13(6): 538–44. 19536422 10.1007/s12603-009-0104-z PMC3100159

[70] Franklin BA, Brinks J, Sacks R, Trivax J, Friedman H. Reduced walking speed and distance as harbingers of the approaching grim reaper. Am J Cardiol. 2015;116(2): 313–7. doi: 10.1016/j.amjcard.2015.04.024 25972052 10.1016/j.amjcard.2015.04.024

[71] Menant JC, Schoene D, Sarofim M, Lord SR. Single and dual task tests of gait speed are equivalent in the prediction of falls in older people: a systematic review and meta-analysis. Ageing Res Rev. 2014;16: 83–104. doi: 10.1016/j.arr.2014.06.001 24915643 10.1016/j.arr.2014.06.001

[72] Muir-Hunter SW, Wittwer JE. Dual-task testing to predict falls in community-dwelling older adults: a systematic review. Physiotherapy. 2016;102(1): 29–40. doi: 10.1016/j.physio.2015.04.011 26390824 10.1016/j.physio.2015.04.011

[73] Corrigan R, McBurney H. Community ambulation: environmental impacts and assessment inadequacies. Disabil Rehabil. 2008;30(19): 1411–9. doi: 10.1080/09638280701654542 18720122 10.1080/09638280701654542

[74] Bohannon RW, Williams Andrews A. Normal walking speed: a descriptive meta-analysis. Physiotherapy. 2011;97(3): 182–9. doi: 10.1016/j.physio.2010.12.004 21820535 10.1016/j.physio.2010.12.004

[75] Salbach NM, O'Brien K, Brooks D, Irvin E, Martino R, Takhar P, et al Speed and distance requirements for community ambulation: a systematic review. Arch Phys Med Rehabil. 2014;95(1): 117–28 e11. doi: 10.1016/j.apmr.2013.06.017 23820298 10.1016/j.apmr.2013.06.017

[76] Chang YK, Pan CY, Chen FT, Tsai CL, Huang CC. Effect of resistance-exercise training on cognitive function in healthy older adults: a review. J Aging Phys Act. 2012;20(4): 497–517. 22186664 10.1123/japa.20.4.497

[77] Voelcker-Rehage C, Godde B, Staudinger UM. Cardiovascular and coordination training differentially improve cognitive performance and neural processing in older adults. Front Hum Neurosci. 2011;5: 26 doi: 10.3389/fnhum.2011.00026 21441997 10.3389/fnhum.2011.00026 PMC3062100

